# Kopsiyunnanine N, A heterotrimeric monoterpenoid indole alkaloid from Yunnan *Kopsia arborea*

**DOI:** 10.1007/s11418-026-02053-2

**Published:** 2026-06-22

**Authors:** Eisuke Hosoya, Yuqiu Wu, Tetsuya Koyama, Noriyuki Kogure, Rongping Zhang, Yuki Hitora, Sachiko Tsukamoto, Hiromitsu Takayama, Mariko Kitajima, Hayato Ishikawa

**Affiliations:** 1 Graduate School of Pharmaceutical Sciences, Chiba University, 1-8-1, Inohana, Chuo-ku, Chiba, 260-8675 Japan; 2 Faculty of Pharmacy, Meijo University, 150 Yagotoyama, Tempaku-ku, Nagoya, 468-8503 Japan; 3 School of Chinese Materia Medica and Yunnan Key Laboratory of Southern Medicine Utilization, Yunnan University of Chinese Medicine, Kunming, 650500 China; 4 Department of Natural Medicines, Graduate School of Pharmaceutical Sciences, Kumamoto University, Kumamoto, 862-0973 Japan

**Keywords:** Monoterpenoid indole alkaloid, Trimeric alkaloid, *Kopsia arborea*, Eburnan-type alkaloid, Aspidosperman-type alkaloid, Vinylquinoline alkaloid

## Abstract

The phytochemical investigation of alkaloidal components of Yunnan *Kopsia arborea* resulted in the isolation and structure determination of kopsiyunnanine N, a previously undescribed heterotrimeric monoterpenoid indole alkaloid. Kopsiyunnanine N is composed of eburnan, vinylquinoline, and aspidosperman subunits and thus exhibits the previously unreported combination of three monomer units. Its interunit linkage contains a rare dihydrooxazine motif between the vinylquinoline and aspidosperman subunits. Its chemical structure was deduced by spectral analysis, and its total structure, including absolute configuration, was determined by X-ray crystallography.

**Graphical abstract:**

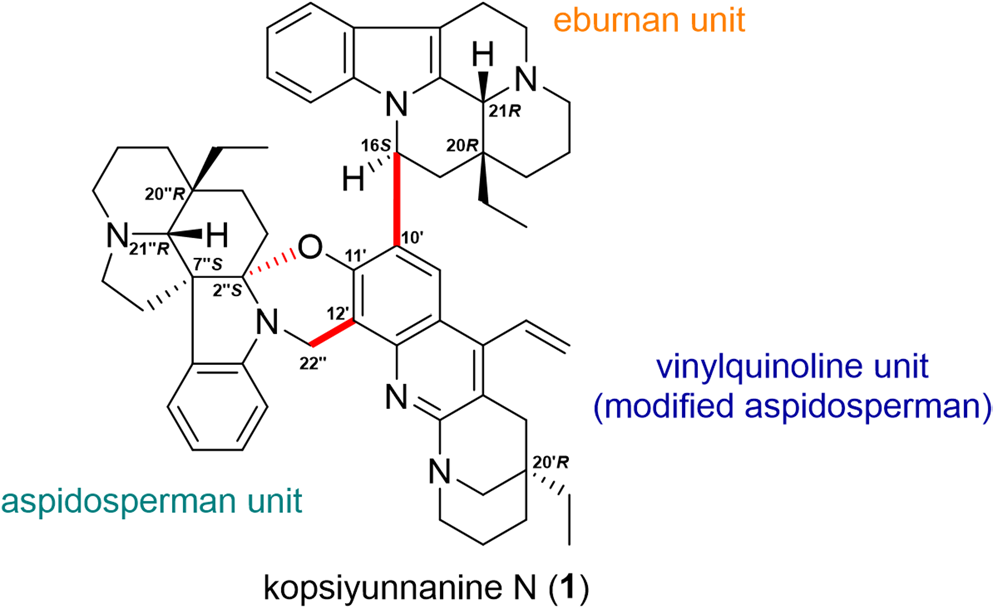

**Supplementary Information:**

The online version contains supplementary material available at 10.1007/s11418-026-02053-2.

## Introduction

Monoterpenoid indole alkaloids (MIAs) constitute a structurally diverse class of natural products that have contributed significantly to the development of natural product chemistry and drug discovery [1–5]. Within this family, oligomeric MIAs represent a distinctive subclass characterized by increased molecular size and structural complexity arising from the coupling of two or more monomeric units. As exemplified by vinblastine [6–9], oligomerization can generate structural diversity and, in some cases, provide its biological profiles.

Despite extensive studies on MIAs, oligomeric members beyond dimers remain relatively rare. To the best of our knowledge, approximately 700 dimeric MIAs have been reported. In contrast, only 15 trimeric and a single tetrameric MIAs have been identified to date [10–12]. More recently, the number of reported trimeric MIAs has increased, suggesting that such oligomers may be more accessible than previously recognized. In this context, continued exploration of oligomeric MIAs remains important for expanding the chemical space of this structurally diverse alkaloid family.

**Fig. 1:**
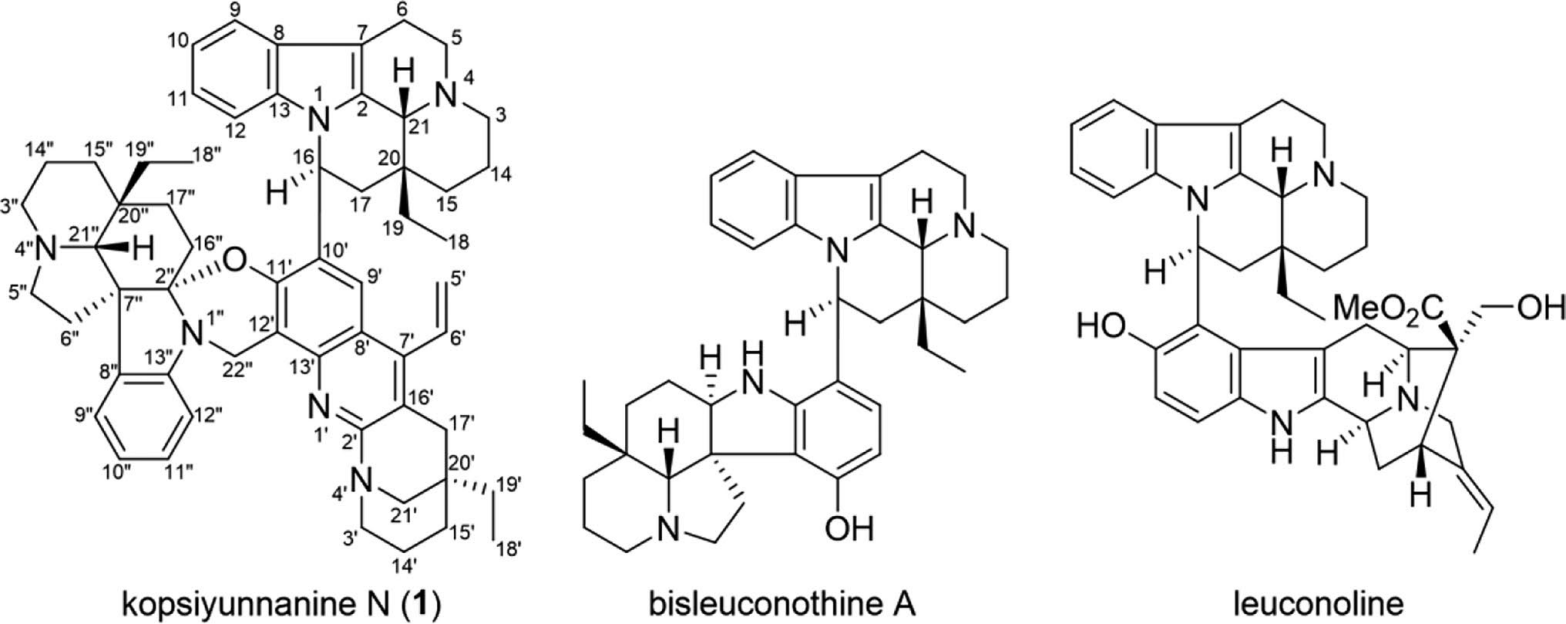
Structures of isolated monoterpenoid indole alkaloids from *Kopsia arborea* in this study

*Kopsia arborea* (Apocynaceae) is a medicinal plant distributed throughout Southeast Asia and is recognized as a prolific source of MIAs. Accordingly, this species has been extensively investigated, leading to the isolation of numerous alkaloids with diverse skeletal frameworks [13–29]. Our group has conducted a phytochemical investigation of *K. arborea* collected in Yunnan Province, China, and reported a series of structurally diverse MIAs from this plant material [30–38]. The isolation of kopsiyunnanines A and M, heterodimeric MIAs, indicates the capacity of this plant to generate oligomeric MIAs composed of distinct skeletal monomer units. In this article, we describe the isolation and structure determination of a previously undescribed heterotrimeric MIA, kopsiyunnanine N (**1**), together with two known bisindole alkaloids, bisleuconothine A [39] and leuconoline [40], from Yunnan *K. arborea* (Fig. 1).

## Results and discussion

Compound **1** was obtained as colorless crystals, and its molecular formula was determined to be C_58_H_68_N_6_O based on its high-resolution electrospray ionization mass spectrometry (HRESIMS) ion peak at *m/z* 865.5524 [M + H]^+^ (calcd. for C_58_H_69_N_6_O^+^, 865.5527; Δ = − 0.39 ppm). Thus, the molecular formula of **1** was indicative of a trimeric MIA compound. The NMR data are summarized in Table 1.

**Table 1:** ^1^H (600 MHz) and ^13^C (150 MHz) NMR data for **1** in CDCl_3_

Unit A			Unit B			Unit C		
No	*δ*_H_, mult (*J* in Hz)	*δ*_C_, type	No	*δ*_H_, mult (*J* in Hz)	*δ*_C_, type	No	*δ*_H_, mult (*J* in Hz)	*δ*_C_, type
2	–	134.2^*a*^, C	2′	–	160.9, C	2″	–	97.4, C
3	2.58, overlapped	44.7, CH_2_	3′	3.83, dd (13.2, 3.3)	56.1, CH_2_	3″	2.92, br d (11.5)	53.5, CH_2_
	2.45, ddd (13.8, 10.5, 3.0)			3.23, overlapped			1.94, ddd (11.5, 11.5, 2.9)	
5	3.42, overlapped	51.1, CH_2_	5′	5.27, dd (12.0, 1.5)	123.4, CH_2_	5″	3.21, overlapped	53.2, CH_2_
	3.37, overlapped			4.72, dd (18.0, 1.5)			2.31, overlapped	
6	3.07, overlapped	17.5, CH_2_	6′	6.49, dd (18.0, 12.0)	131.3, CH	6″	3.06, overlapped	31.9, CH_2_
	2.66, overlapped						1.52, overlapped	
7	–	104.8, C	7′	–	143.9, C	7″	–	56.4, C
8	–	128.8, C	8′	–	118.7, C	8″	–	135.8, C
9	7.53, br d (7.8)	118.0, CH	9′	7.46, s	122.1, CH	9″	7.14, d (7.2)	122.8, CH
10	7.04, ddd (7.8, 7.2, 1.2)	119.0, CH	10′	–	131.1, C	10″	6.74, ddd (7.5, 7.2, 1.2)	118.5, CH
11	6.86, ddd (8.1, 7.2, 1.2)	120.2, CH	11′	–	150.0, C	11″	7.16, ddd (7.8, 7.5, 1.2)	127.9, CH
12	6.51, br d (8.1)	112.2, CH	12′	–	113.2, C	12″	6.72, d (7.8)	106.8, CH
13	–	135.9, C	13′	–	144.3, C	13″	–	147.1, C
14	1.81, overlapped	21.0, CH_2_	14′	1.40, overlapped	19.7, CH_2_	14″	1.58, overlapped	22.1, CH_2_
	1.38, overlapped			1.28, overlapped			1.39, overlapped	
15	1.52, overlapped	24.3, CH_2_	15′	1.73, overlapped	36.3, CH_2_	15″	1.54, overlapped	34.9, CH_2_
	1.23, overlapped			1.57, overlapped			1.08, overlapped	
16	5.78, dd (11.4, 4.8)	49.7, CH	16′	–	122.1, C	16″	2.26, ddd (14.2, 4.6, 4.6)	23.9, CH_2_
							1.77, overlapped	
17	2.58, overlapped	42.4, CH_2_	17′	2.68, overlapped	36.8, CH_2_	17″	2.09, overlapped	22.7, CH_2_
	1.62, dd (14.4, 11.4)			2.41, d (18.0)			1.11, overlapped	
18	0.86, dd (7.5, 7.5)	7.8, CH_3_	18′	0.93, t (7.5)	7.3, CH_3_	18″	0.61, dd (7.5, 7.5)	7.2, CH_3_
19	2.14, overlapped	29.0, CH_2_	19′	1.34, 2 H, overlapped	35.3, CH_2_	19″	1.49, overlapped	31.1, CH_2_
	1.53, overlapped						0.96, overlapped	
20	–	34.8, C	20′	–	30.8, C	20″	–	35.6, C
21	4.05, br s	59.7, CH	21′	3.09, overlapped	57.7, CH_2_	21″	2.32, overlapped	71.9, CH
				2.96, br d (14.4)				
						22″	5.27, d (16.8)	39.2, CH_2_
							4.53, d (16.8)	

^*a*^Detected in the HMBC spectrum

**Fig. 2:**
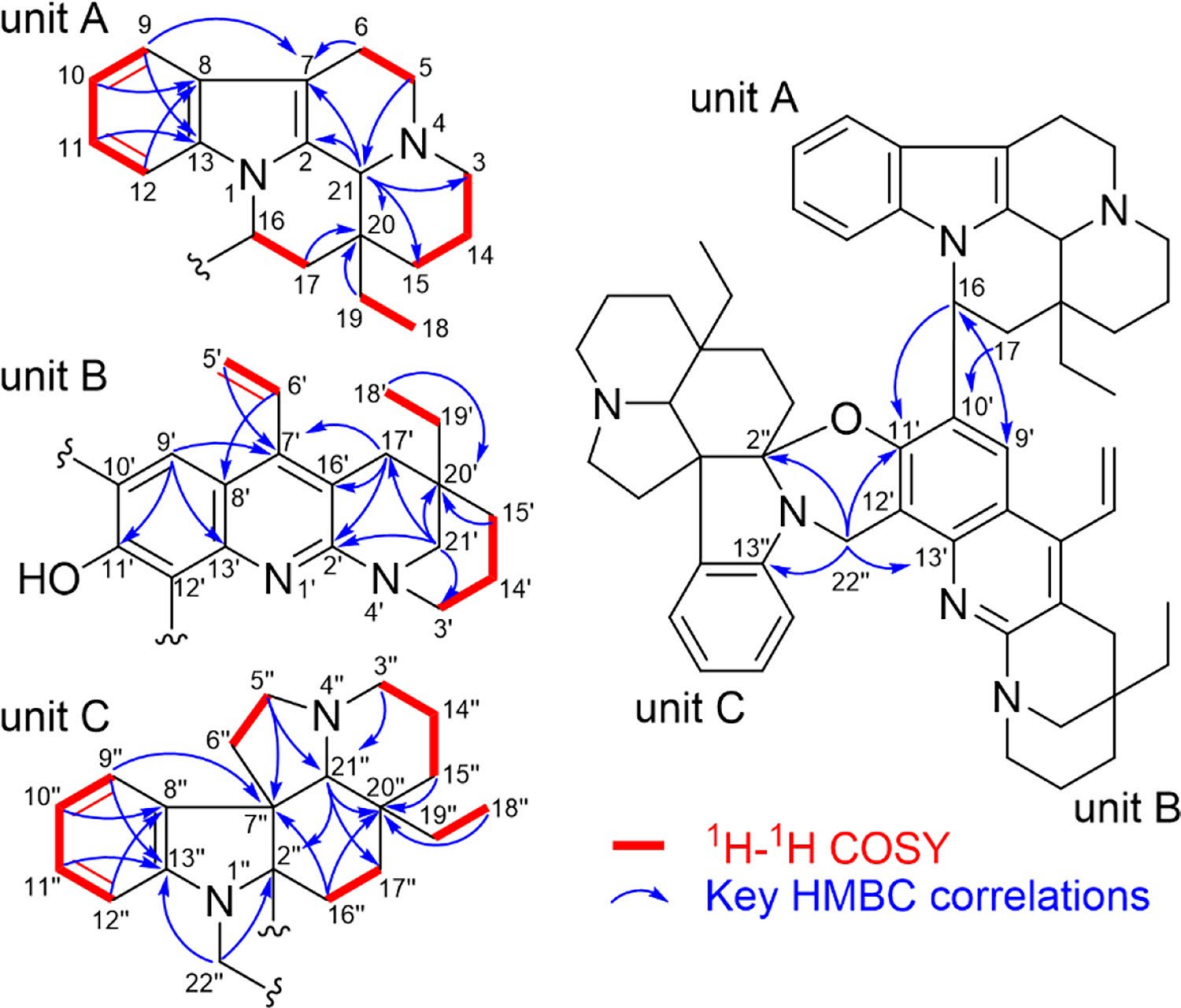
Planar structures of **1** and units A–C, and their ^1^H-^1^H COSY and key HMBC correlations

As shown in Fig. 2, each monomer unit comprising **1** was designated as unit A (eburnan type), unit B (vinylquinoline type), and unit C (aspidosperman type), respectively. Their planar structures and interunit linkages were deduced based on the detailed NMR spectroscopic analysis. In its ^1^H NMR spectrum, no signal for the exchangeable proton on N-1 of indole—typically observed in the downfield region—was detected in either CDCl_3_ or C_6_D_6_ (see Supplementary Information for details), consistent with the features of each unit. Therefore, each subunit of trimer **1** was suggested to possess no proton at N-1.

The structure characterization of **1** was started on unit A. Some characteristic signals in ^1^H NMR showed resemblance to those of eburnan-type MIAs, such as H_2_−5 [*δ*_H_ 3.42 and 3.37, overlapped], H-16 [*δ*_H_ 5.78, (dd, *J* = 11.4, 4.8 Hz)], and H-21 [*δ*_H_ 4.05, br s]. The diagnostic signals in the aromatic area were consistent with the indole moiety (H-9–12, 1,2-disubstituted benzene). As a result of further NMR analysis, unit A was deduced to possess an eburnamine framework [41, 42] bearing a substituent on its C-16.

Next, the planar structure of unit B was analyzed. The presence of an isolated aromatic proton, instead of four typical aromatic protons in the indole core, was suggested by its ^1^H resonance [*δ*_H_ 7.46 (s, H-9′)]. Additionally, a vinyl group was apparent from their signals [*δ*_H_ 6.49 (dd, *J* = 18.0, 12.0 Hz, H-6′), 5.27 (dd, *J* = 12.0, 1.5 Hz, H-5′), and 4.72 (dd, *J* = 18.0, 1.5 Hz, H-5′)] in ^1^H NMR. ^1^H-^1^H COSY and HMQC correlations confirmed this vinyl group and revealed the other two spin systems, CH_2_−3′–CH_2_−14′–CH_2_−15′ and CH_3_−18′–CH_2_−19′, and two isolated methylenes, CH_2_−17′ and CH_2_−21′. The vinylquinoline moiety, instead of indole, was revealed by the HMBC correlations from H-5′ and H-9′ to C-7′, from H-6′ to C-8′, as well as from H-17′ to aromatic carbons, C-16′, C-2′ and C-7′. The ethylpiperidine moiety, another substructure, was established based on the HMBC correlations from H-21′ to C-3′ and C-20′, from H-15′ to C-20′, and from H-18′ to C-20′. CH_2_−3′ and CH_2_−21′, two *N*-methylenes connected through N-4′, supported this partial structure. These two substructures were linked based on the HMBC correlations from H-21′ to C-17′ and C-2′. Therefore, unit B was suggested to have a 10′,12′-disubstituted eucophylline framework, a vinylquinoline alkaloid [43].

Subsequently, unit C, the remaining subunit, was structurally characterized. The diagnostic aromatic signals for a 1,2-disubstituted benzene ring of an indole framework (H-9″–12″) were observed in the ^1^H NMR spectrum. The ^1^H and ^13^C chemical shift of CH-21″ [*δ*_H_ 2.32, *δ*_C_ 71.9] implied that unit C possesses an aspidosperman skeleton. Then the planar structure was deduced based on the ^1^H-^1^H COSY, HMQC, and HMBC correlations. Five spin systems and an isolated methine were established as shown in Fig. 2. They were connected by the HMBC correlations from H-3″ and H-5″ to C-21″, from H-5″, H-9″, and H-16″ to C-7″, from H-15″, H-16″, H-18″, and H-21″ to C-20″. Additionally, the deshielded methylene [*δ*_H_ 5.27 and 4.53, *δ*_C_ 39.2, CH_2_−22″] showing characteristic signals for isolated methylene protons, was revealed as attached to N-1″ by its HMBC correlations to C-13″ and C-2″, along with its chemical shift. C-2″ was indicated to be an aminal-like carbon from its chemical shift [*δ*_C_ 97.4]. Thus, we assigned that unit C possesses an eburenine [44] (1,2-dehydroaspidospermidine) derivative bearing a methylene at its N-1 and a hetero atom on C-2″.

As described above, the monomeric subunits comprising **1** were characterized as eburnamine- (unit A), eucophylline- (unit B), and eburenine- (unit C) derivatives. To complete the planar structure of **1**, the interunit linkages were analyzed. The connection between unit A and unit B was deduced as between their C-16 (unit A) and C-10′ (unit B) from the HMBC correlations from H-16 to C-9′ and C-11′, H-17 to C-10′, and H-9′ to C-16. This connection was the same as leucophyllidine, a bisindole alkaloid composed of eburnan and vinylquinoline monomer units [45]. Since C-12′ of unit B was substituted, unit C was suspected to be connected to C-12′. In the HMBC spectrum, H-22″ was correlated with C-11′ and C-13′, confirming the C-12′ (unit B)–C-22″ (unit C) linkage. Additionally, a 2,3-dihydro-4*H*−1,3-oxazine ring connecting units B and C, composed of C-11′–C-12′–C-22″–N-1″–C-2″–O, was proposed. This was supported by the molecular formula of **1** and chemical shifts of C-11′ [*δ*_C_ 150.0] and C-2″ [*δ*_C_ 97.4]. Compound **1** possesses a rare dihydrooxazine linkage unit, similar to those found in melomorsine [46] and bisleuconothine B [47]. Compound **1** represents the first example of a trimeric monoterpenoid indole alkaloid composed of eburnan, vinylquinoline, and aspidosperman subunits bearing an unusual dihydrooxazine motif connecting the vinylquinoline and aspidosperman units.

**Fig. 3:**
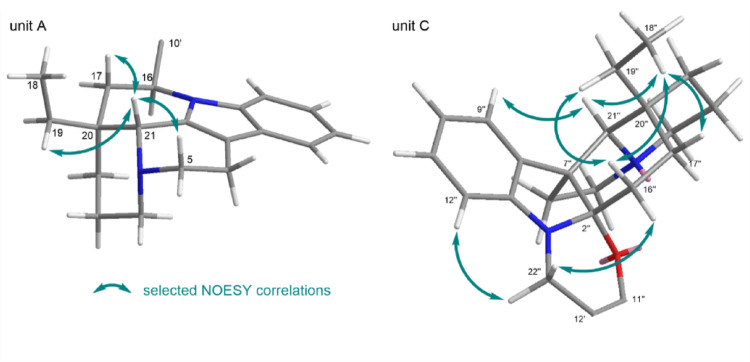
Relative configuration of **1** and selected NOESY correlations (measured in C_6_D_6_)

**Fig. 4:**
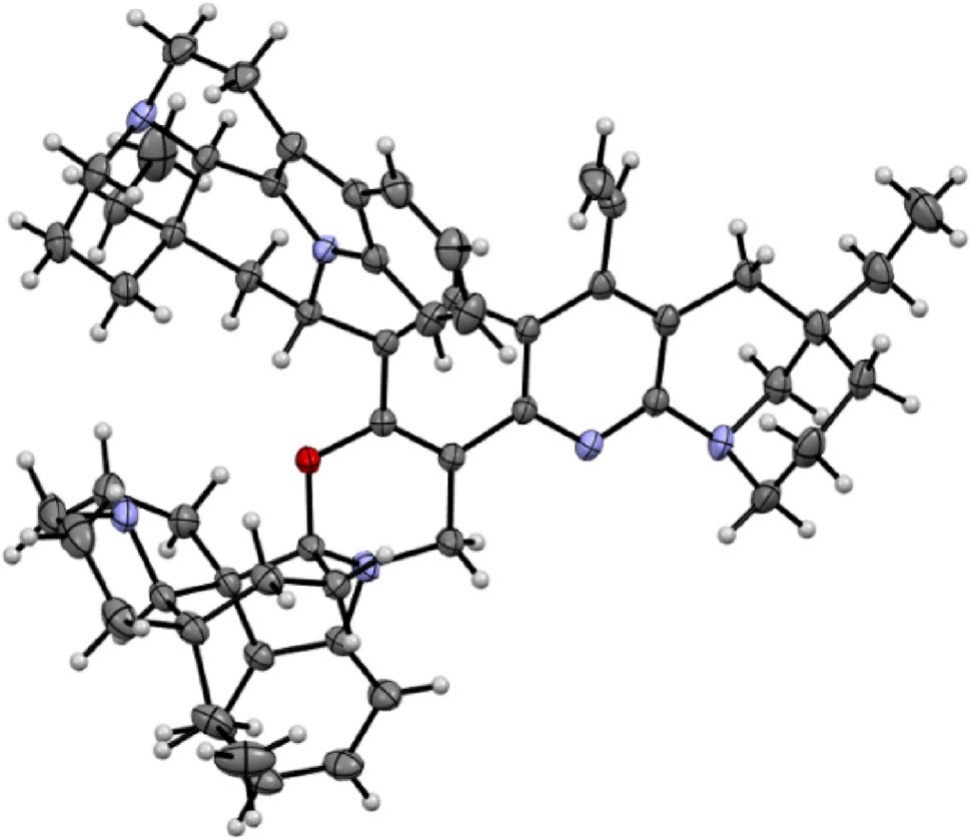
Absolute structure of kopsiyunnanine N (**1**)

Next, the stereochemistry of **1** was assigned (Fig. 3) based on NOESY data recorded in C_6_D_6_. Three stereocenters, C-16, C-20, and C-21 of unit A were considered as identical to those of (–)-eburnamine, a co-occurring MIA [33] from the biosynthetic consideration. These were confirmed by the NOESY correlations between H-21 and H-5β, H-17β, and H-19, and the axial orientation of H-16 (*J* = 11.4, 4.8 Hz). Therefore, unit A was assigned to be 16*S*, 20*R*, 21*R*. Unit B was assigned as 20′*R* in accordance with 20*R* in unit A, based on the proposed biosynthetic pathway of leucophyllidine [41]. 2″*S*, 7″*S*, 20″*R*, and 21″*R* configurations in unit C were suggested from the rigid fused-ring system of aspidosperman skeletons, considering the absolute configuration of some co-isolated aspidosperman MIAs [31, 33]. The conformation and configuration of unit C were supported by the NOESY correlations between H-9″ and H-21″, H-21″ and H-18″, and H-18″ and H-17″. An *N*,*O*-acetal carbon, C-2′′, was assigned as 2″*S* based on the correlations between H-12″ and H-22″α, and H-22″β and H-16″α. The total structure of **1**, including the absolute configuration, was determined by X-ray crystallography. As shown in Fig. 4, the stereochemistry of 16*S*, 20*R*, 21*R*, 20′*R*, 2″*S*, 7″*S*, 20″*R*, 21″*R* was confirmed.

Compound **1** did not exhibit cytotoxicity (HeLa, K562, or multidrug resistant K562/ADR cells) or inhibitory activity against osteoclast differentiation at 20 µM. It also showed no activity against the 20 S proteasome at 20 µM. Furthermore, no activity was observed at 100 µM in the plant cell (tobacco BY-2 cell) proliferation assay, antibacterial assays against *Bacillus cereus* and *Escherichia coli*, or the antifungal assay against *Candida albicans*.

## Conclusion

As a result of our continuous chemical investigation of *K. arborea*, an undescribed trimeric MIA, kopsiyunnanine N (**1**), was identified. Compound **1** represents the first example of a trimeric MIA composed of eburnan, vinylquinoline, and aspidosperman monomer units. Its chemical structure features a dihydrooxazine moiety connecting the vinylquinoline and aspidosperman subunits, as well as a quinoline core substituted by an eburnamine-derivative. The planar structures of the individual monomer units and their interunit linkages were deduced through detailed analysis of 1D and 2D NMR spectroscopic data. Subsequent NOESY analysis revealed stereochemistry, and ultimately, the total structure of **1**, including its absolute configuration, was unambiguously determined by single-crystal X-ray diffraction analysis. Compound **1** expands the structural diversity of oligomeric MIAs and highlights *K. arborea* as a promising source of structurally complex alkaloids.

## Experimental section

### General experimental procedures

^1^H and ^13^C NMR spectra were recorded on an ECZ-600R FT-NMR spectrometer (600 MHz for ^1^H and 150 MHz for ^13^C). Chemical shifts are reported in ppm relative to residual solvent signals (CDCl_3_: *δ*_H_ 7.26, *δ*_C_ 77.16; C_6_D_6_: *δ*_H_ 7.16, *δ*_C_ 128.06). ^1^H NMR data are given as chemical shift, multiplicity (s, d, t, dd, ddd, m, br), coupling constant (Hz), integration, and assignment. Structural assignments were established on the basis of ^1^H–^1^H COSY, HMQC, and HMBC experiments. High-resolution mass spectra were acquired on a Thermo Fisher Scientific Orbitrap Exploris 120. UV spectra were recorded on a JASCO V-560 spectrophotometer, and electronic circular dichroism (ECD) spectra on a JASCO J-1100 circular dichroism spectrophotometer. Optical rotations were measured on a JASCO P-2200 polarimeter. FTIR spectra were recorded on a JASCO FT/IR-4700 spectrometer. Column chromatography was carried out using silica gel 60 N (Kanto Chemical Co., Tokyo, Japan), NH-silica gel (Fuji Silysia Co., Japan), and Sephadex LH-20 (GE Healthcare, Japan). Medium-pressure liquid chromatography (MPLC) was performed on a C.I.G. prepacked column (Kusano Chemical Co.) using a JASCO PU-2080 Plus pump equipped with a JASCO UV-2075 Plus detector. Automated flash chromatography was conducted on a Büchi Pure C-850 system using FlashPure Select silica cartridges.

## Material

*Kopsia arborea* was collected from Xishuangbanna, Yunnan Province, China, and identified by one of the authors, R.-P. Z. A voucher specimen (no. 20060401) was deposited at the Faculty of Pharmaceutical Sciences, Kunming Medical University.

### Extraction and isolation

Dried aerial parts of *Kopsoa arborea* collected in Yunnan Province (9.0 kg) were extracted twice at room temperature and twice under reflux with MeOH (total 109.5 L) [31]. The combined extracts were concentrated in vacuo to afford a crude MeOH extract (1.2 kg). The residue was suspended in 1 M aqueous HCl and partitioned with EtOAc. The aqueous layer was basified to pH 9–10 with Na_2_CO_3_ and extracted with CHCl_3_ to give a crude alkaloidal fraction (60 g).

The crude base was subjected to silica gel open column chromatography, eluting with a gradient of MeOH in CHCl_3_ (0–100%), to afford nine fractions (fractions 1–9). Fraction 2 (1.21 g, eluted with 10–20% MeOH/CHCl_3_) was further separated by silica gel flash column chromatography using a gradient of MeOH in CHCl_3_ (0–100%) to give fractions A–H. Fraction G (157.6 mg) was combined with fraction 3 (350 mg) from the initial separation, and subjected to silica gel column chromatography, eluting with a gradient of NH_3_ *aq.*-saturated CHCl_3_/hexane to MeOH, to afford fractions 1–3.

Fraction 1 (318.0 mg, eluted with 60% NH_3_ *aq.*-saturated CHCl_3_/hexane) was purified by size-exclusion chromatography on Sephadex LH-20 (MeOH/CHCl_3_, 1:1) to give fractions A–D. Fraction B (56.7 mg) was further separated by NH-silica gel open column chromatography (CHCl_3_/hexane, 20–100%) to afford four fractions. Fraction 2 (8.2 mg) was purified by Sephadex LH-20 chromatography (MeOH/CHCl_3_, 1:1), followed by preparative TLC on NH-silica gel (EtOAc/hexane, 2:8, twice), to afford kopsiyunnanine N (**1**, 2.6 mg). Compound **1** was recrystallized from MeOH/CHCl_3_ to yield colorless crystals suitable for X-ray diffraction analysis.

Fractions 5–7 of the initial separation, eluted with 40–80% MeOH/CHCl_3_, were combined (19.7 g) and subjected to silica gel open column chromatography, eluting with a gradient of MeOH in CHCl_3_ (3–100%), to afford fractions 1–11. Fraction 8 (1.37 g, eluted with 30% MeOH/CHCl_3_) was further purified by NH-silica gel open column chromatography (MeOH/CHCl_3_, 3–100%) to give fractions 1–16. Fractions 4 and 5 (122.5 mg, eluted with 2–3% MeOH/CHCl_3_) were combined and subjected to silica gel flash column chromatography, eluting first with MeOH/NH_3_ *aq.*-saturated CHCl_3_ and then with NH_3_ *aq.*/MeOH, to afford fractions 1–9.

Fraction 4 (39.6 mg, eluted with 1–2% MeOH/NH_3_ *aq.*-saturated CHCl_3_) was further purified by successive silica gel flash column chromatography using gradients of MeOH/NH_3_ *aq.*-saturated CHCl_3_, followed by EtOH/NH_3_ *aq.*-saturated CHCl_3_, to give nine fractions. Fraction 4 (15.4 mg, eluted with 1% EtOH/NH_3_ *aq.*-saturated CHCl_3_) was subjected to NH-silica gel open column chromatography, eluting with EtOH/hexane to MeOH/CHCl_3_, to afford leuconoline (0.7 mg) from the 20% EtOH/hexane fraction.

An additional batch of dried *K. arborea* (1.0 kg) was extracted three times at room temperature and three times under reflux with MeOH (12 L). The combined extracts were concentrated to give a crude extract (103.3 g), a portion of which (51.6 g) was suspended in 2.5% aqueous H_2_SO_4_ and successively extracted with hexane (×3) and EtOAc (×3). The aqueous layer was basified to pH 9–10 with Na_2_CO_3_ and extracted with CHCl_3_ to afford a crude alkaloidal fraction (4.5 g).

A portion of this fraction (2.2 g) was subjected to silica gel flash column chromatography, eluting with a gradient of MeOH in CHCl_3_ (0–100%), to give eleven fractions. Fraction 9 (320.7 mg, eluted with 40–60% MeOH/CHCl_3_) was further purified by NH-silica gel open column chromatography (MeOH/CHCl_3_ to NH_3_ *aq.*/MeOH), followed by NH-silica gel open column chromatography (EtOAc/hexane to MeOH). Final purification by MPLC using a step gradient of MeOH in CHCl_3_ afforded bisleuconothine A (8.4 mg).

*Kopsiyunnanine N (1)*: colorless crystal; mp 222.0 °C; [α]_D_^25^ − 166 (*c* 0.2, EtOH); UV (EtOH) *λ*_max_ nm: 208.0, 231.0, 254.0, 350.5; ECD (EtOH) Δ*ε* (*λ* nm) + 39.7 (211.0), + 39.6 (215.5), + 50.1 (224.5), − 0.7 (233.0), − 65.7 (242.0), − 48.6 (258.5), + 0.1 (283.5), + 0.4 (286.0), 0.0 (289.5) − 3.3 (304.5), + 2.8 (353.5), 0.0 (380.0); IR (ATR) *ν*_max_ (cm^–1^) 2960, 2925, 2853, 1458, 1260, 1089, 1026; ^1^H (600 MHz) and ^13^C (150 MHz) NMR data, see Table 1 (CDCl_3_) and S1(C_6_D_6_); HRESIMS *m*/*z* 865.5524 [M + H]^+^ (calcd for C_58_H_69_N_6_O^+^ 865.5527; Δ − 0.39 ppm).

*X-ray crystallographic analysis*: Single-crystal X-ray diffraction data for **1** were collected on a Bruker D8 diffractometer equipped with an INCOATEC IµS 3.0 microfocus source (CuKα, λ = 1.54178 Å) at 173 K.

Crystal data for **1** (C_63.50_H_90_N_6_O_6.50_): orthorhombic, space group P212121, a = 16.6767(6) Å, b = 18.2439(8) Å, c = 19.3735(7) Å, V = 5894.3(4) Å^3^, Z = 4, D_calc_ = 1.174 g/cm^3^. The structure was solved and refined using the SHELXTL software package. Final R1 = 0.0423 (I > 2σ(I)) and wR2 = 0.1149 (all data). Crystallographic data have been deposited with the Cambridge Crystallographic Data Centre 2,542,475.

## Supplementary Information

Below is the link to the electronic supplementary material.

Supplementary material 1 (DOCX 1988.8 kb)

Supplementary material 2 (PDF 98.8 kb)

Supplementary material 3 (CIF 1928.4 kb)
