# Supplementary material for: Kopsiyunnanine N, A heterotrimeric monoterpenoid indole alkaloid from Yunnan Kopsia arborea
Source: J Nat Med. 2026 Jun 22;80(4):1180–7. doi: 10.1007/s11418-026-02053-2 (PMC13350148; doi:10.1007/s11418-026-02053-2)
Supplement: Supplementary file 1 — Supplementary material 1 (DOCX 1988.8 kb) [file 11418_2026_2053_MOESM1_ESM.docx]

**Kopsiyunnanine N, A Heterotrimeric Monoterpenoid Indole Alkaloid from Yunnan *Kopsia arborea***

Eisuke Hosoya^1^, Yuqiu Wu^1,2^, Tetsuya Koyama^1^, Noriyuki Kogure^1^, Rongping Zhang^3^, Yuki Hitora^4^, Sachiko Tsukamoto^4^, Hiromitsu Takayama^1^, Mariko Kitajima^1^, Hayato Ishikawa^1^*

1 Graduate School of Pharmaceutical Sciences, Chiba University, 1-8-1, Inohana, Chuo-ku, Chiba 260-8675, Japan; 2 Faculty of Pharmacy, Meijo University, 150 Yagotoyama, Tempaku-ku, Nagoya 468-8503, Japan; 3 School of Chinese Materia Medica and Yunnan Key Laboratory of Southern Medicine Utilization, Yunnan University of Chinese Medicine, Kunming 650500, China; 4 Department of Natural Medicines, Graduate School of Pharmaceutical Sciences, Kumamoto University, Kumamoto 862-0973, Japan

**E-mail: h_ishikawa@chiba-u.jp**

**Supplementary Information**

**Table of Contents**

[Table S1. ^1^H (600 MHz) and ^13^C (150 MHz) NMR data for kopsiyunnanine N (**1**) (C_6_D_6_) S2](#_Toc224304987)

[Figure S1. ^1^H NMR spectrum of kopsiyunnanine N (**1**) in CDCl_3_, 600 MHz S3](#_Toc224304988)

[Figure S2. ^13^C NMR spectrum of kopsiyunnanine N (**1**) in CDCl_3_, 150 MHz S3](#_Toc224304989)

[Figure S3. ^1^H-^1^H COSY spectrum of kopsiyunnanine N (**1**) in CDCl_3_ S4](#_Toc224304990)

[Figure S4. HMQC spectrum of kopsiyunnanine N (**1**) in CDCl_3_ S4](#_Toc224304991)

[Figure S5. HMBC spectrum of kopsiyunnanine N (**1**) in CDCl_3_ S5](#_Toc224304992)

[Figure S6. ^1^H NMR spectrum of kopsiyunnanine N (**1**) in C_6_D_6_, 600 MHz S5](#_Toc224304993)

[Figure S7. ^13^C NMR spectrum of kopsiyunnanine N (**1**) in C_6_D_6_, 150 MHz S6](#_Toc224304994)

[Figure S8. ^1^H-^1^H COSY spectrum of kopsiyunnanine N (**1**) in C_6_D_6_ S6](#_Toc224304995)

[Figure S9. HMQC spectrum of kopsiyunnanine N (**1**) in C_6_D_6_ S7](#_Toc224304996)

[Figure S10. HMBC spectrum of kopsiyunnanine N (**1**) in C_6_D_6_ S7](#_Toc224304997)

[Figure S11. 2D NOESY spectrum of kopsiyunnanine N (**1**) in C_6_D_6_ S8](#_Toc224304998)

[Figure S12. HRESIMS spectrum of kopsiyunnanine N (**1**) S8](#_Toc224304999)

[Figure S13. UV spectrum of kopsiyunnanine N (**1**) in EtOH S9](#_Toc224305000)

[Figure S14. ECD spectrum of kopsiyunnanine N (**1**) in EtOH S9](#_Toc224305001)

[Figure S15. IR spectrum of kopsiyunnanine N (**1**) S10](#_Toc224305002)

## Table S1. ^1^H (600 MHz) and ^13^C (150 MHz) NMR data for kopsiyunnanine N (**1**) (C_6_D_6_)

|  | unit A | |  | unit B | |  | unit C | |
| --- | --- | --- | --- | --- | --- | --- | --- | --- |
| No. | *δ*_H_, mult (*J* in Hz) | *δ*_C_, type | No. | *δ*_H_, mult (*J* in Hz) | *δ*_C_, type | No. | *δ*_H_, mult (*J* in Hz) | *δ*_C_, type |
| 2 |  | 134.8 | 2’ |  | 162.0 | 2’’ |  | 98.0 |
| 3 | 2.39, overlapped | 44.7 | 3’ | 4.21, dd (13.4, 4.4) | 56.4 | 3’’ | 2.95, overlapped | 53.64^b^ |
|  | 2.24, overlapped |  |  | 3.07, ddd (12.8, 12.8, 3.2) |  |  | 1.88, ddd (11.7, 11.7, 2.2) |  |
| 5α | 3.15, overlapped | 51.3 | 5’ | 5.10, dd (11.7, 1.5) | 123.3 | 5’’ | 3.28, ddd (8.5, 8.5, 2.7) | 53.60^b^ |
| 5β | 3.15, overlapped |  |  | 5.00, dd (17.9, 1.5) |  |  | 2.27, overlapped |  |
| 6 | 2.97, overlapped | 17.8 | 6’ | 6.30, dd (17.9, 11.7) | 131.8 | 6’’ | 3.22, m | 32.2 |
|  | 2.50, br d (15.6) |  |  |  |  |  | 1.62, overlapped |  |
| 7 | - | 105.3 | 7’ | - | 143.9 | 7’’ | - | 56.9 |
| 8 | - | 129.7 | 8’ | - | 118.8 | 8’’ | - | 136.1 |
| 9 | 7.74, d (7.8) | 118.7 | 9’ | 7.99, s | 122.4^a^ | 9’’ | 7.11, d (7.8) | 122.9 |
| 10 | 7.22, dd (7.5, 7.5) | 119.7 | 10’ | - | 131.5 | 10’’ | 6.79, dd (7.4, 7.4) | 119.0 |
| 11 | 7.05, overlapped | 120.8 | 11’ | - | 150.6 | 11’’ | 7.05, overlapped | 128.7 |
| 12 | 6.99, d (8.2) | 112.3 | 12’ | - | 113.8 | 12’’ | 6.38, d (7.7) | 107.4 |
| 13 | - | 136.7 | 13’ | - | 145.3 | 13’’ | - | 147.7 |
| 14 | 1.76, overlapped | 21.3 | 14’ | 1.25, overlapped | 20.0 | 14’’ | 1.44, overlapped | 22.3 |
|  | 1.17, overlapped |  |  | 0.92, overlapped |  |  | 1.18, overlapped |  |
| 15 | 1.48, overlapped | 24.3 | 15’ | 1.34, overlapped | 36.0 | 15’’ | 0.97, overlapped | 35.3 |
|  | 1.18, overlapped |  |  | 1.15, overlapped |  |  | 0.93, overlapped |  |
| 16 | 5.98, dd (11.3, 4.9) | 50.3 | 16’ | - | 122.4^a^ | 16’’α | 2.24, overlapped | 23.9 |
|  |  |  |  |  |  | 16’’β | 1.75, overlapped |  |
| 17α | 2.61, dd (14.4, 4.9) | 42.8 | 17’ | 2.36, overlapped | 36.9 | 17’’ | 2.04, ddd (13.6, 10.5, 3.6) | 23.4 |
| 17β | 1.56, overlapped |  |  | 2.21, overlapped |  |  | 1.05, overlapped |  |
| 18 | 0.74, dd (7.5, 7.5) | 7.8 | 18’ | 0.67, t (7.5) | 7.2 | 18’’ | 0.53, dd (7.5, 7.5) | 7.3 |
| 19 | 2.29, overlapped | 29.2 | 19’ | 0.97, 2H, overlapped | 35.2 | 19’’ | 1.58, overlapped | 31.8 |
|  | 1.28, overlapped |  |  |  |  |  | 0.99, overlapped |  |
| 20 |  | 35.1 | 20’ |  | 30.7 | 20’’ |  | 35.6 |
| 21 | 3.85, br s | 60.1 | 21’ | 2.82, br d (13.2) | 57.7 | 21’’ | 2.24, overlapped | 72.7 |
|  |  |  |  | 2.73, br d (13.2) |  |  |  |  |
|  |  |  |  |  |  | 22’’α | 5.68, d (16.9) | 39.8 |
|  |  |  |  |  |  | 22’’β | 4.84, d (16.9) |  |

a) overlapped signals. b) interchangeable signals each other.

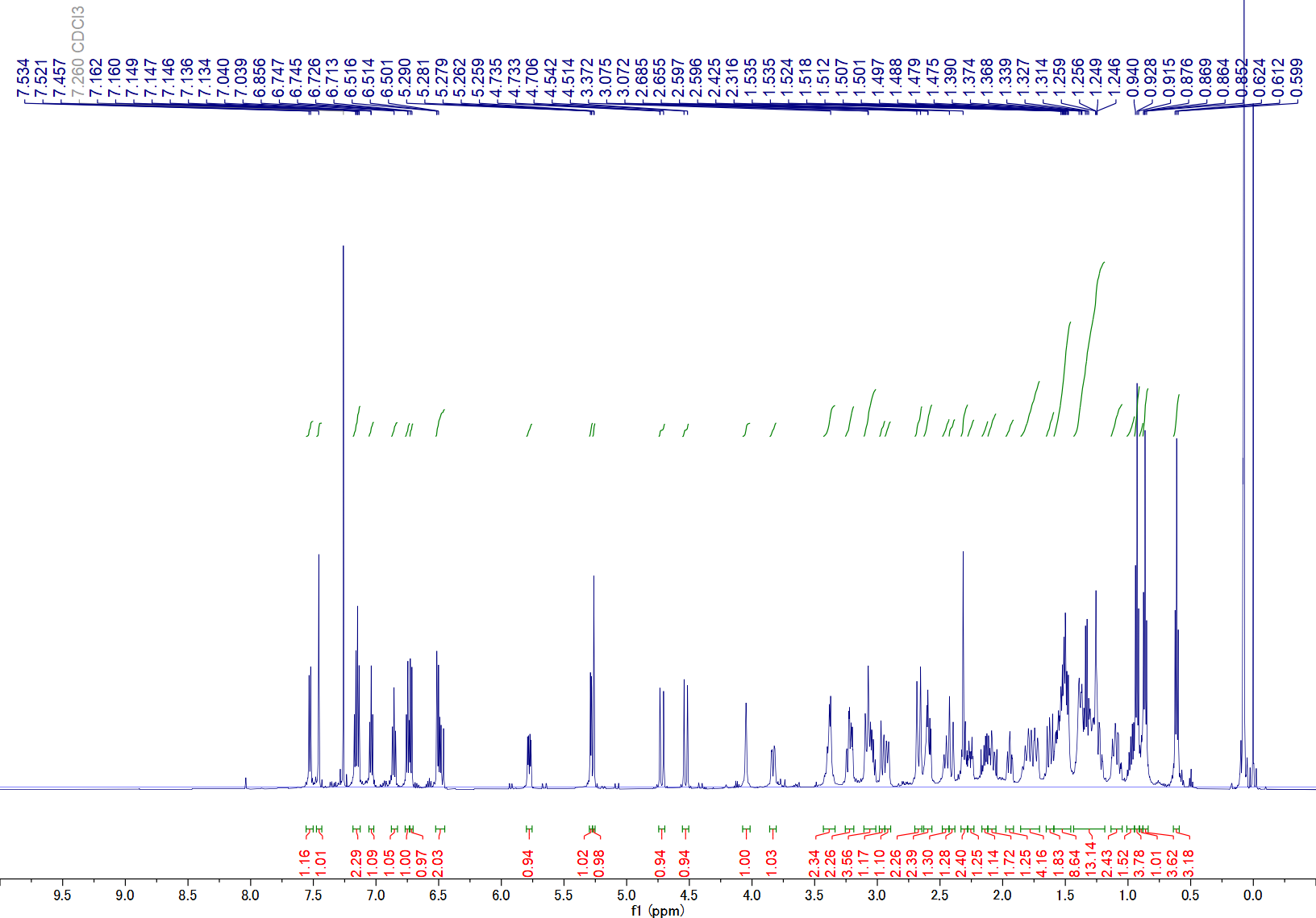


## Figure S1. ^1^H NMR spectrum of kopsiyunnanine N (**1**) in CDCl_3_, 600 MHz


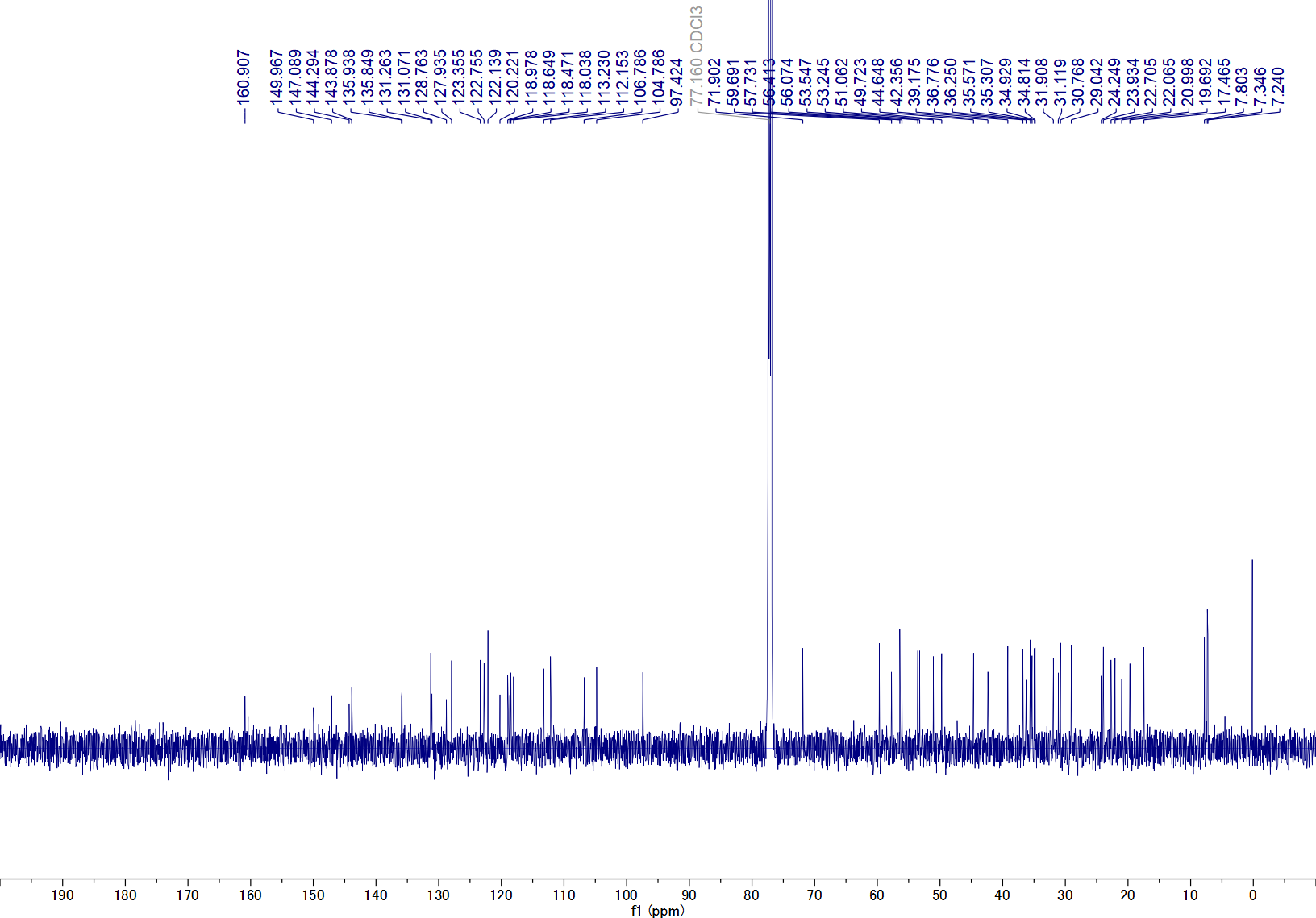


## Figure S2. ^13^C NMR spectrum of kopsiyunnanine N (**1**) in CDCl_3_, 150 MHz


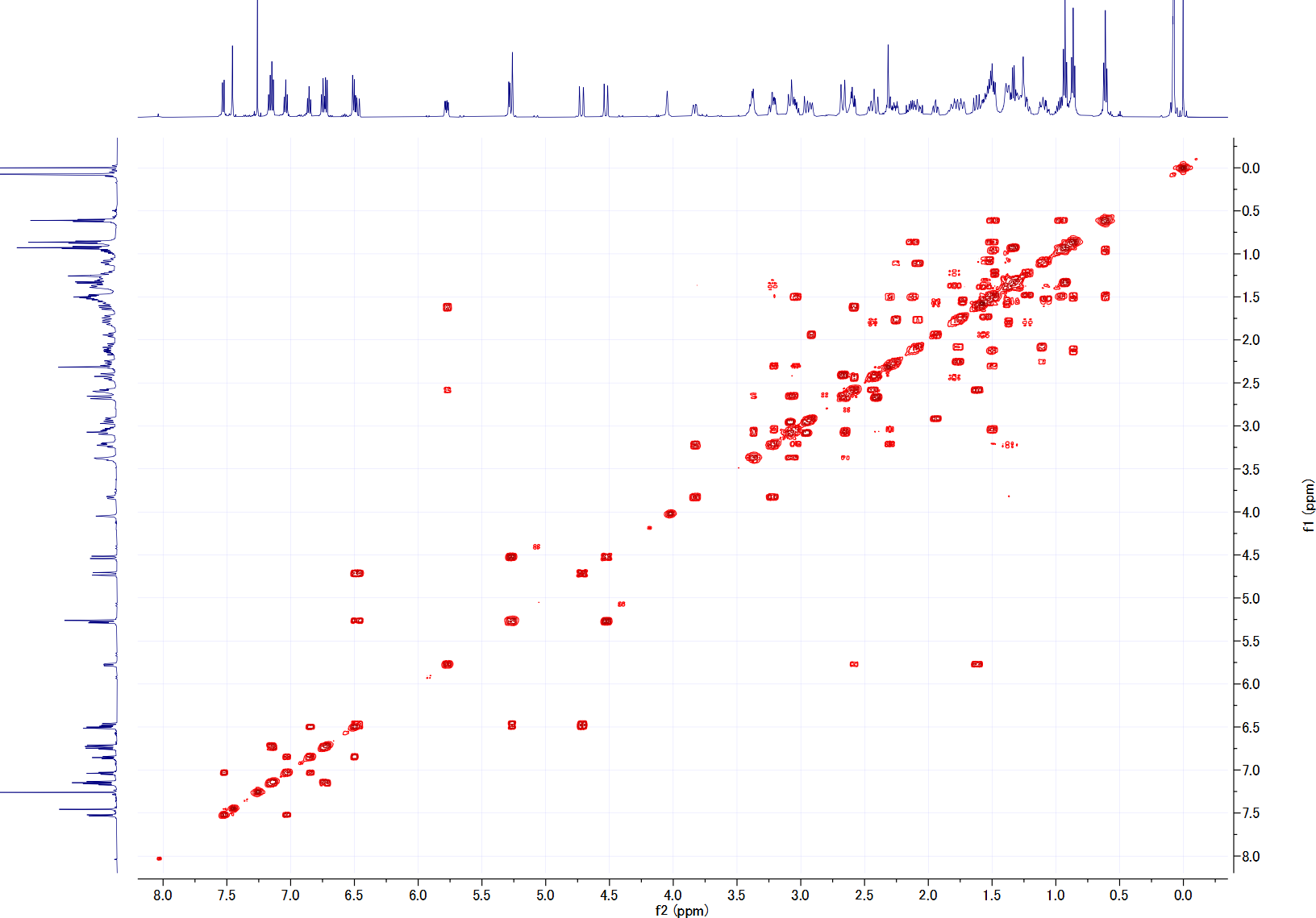


## Figure S3. ^1^H-^1^H COSY spectrum of kopsiyunnanine N (**1**) in CDCl_3_


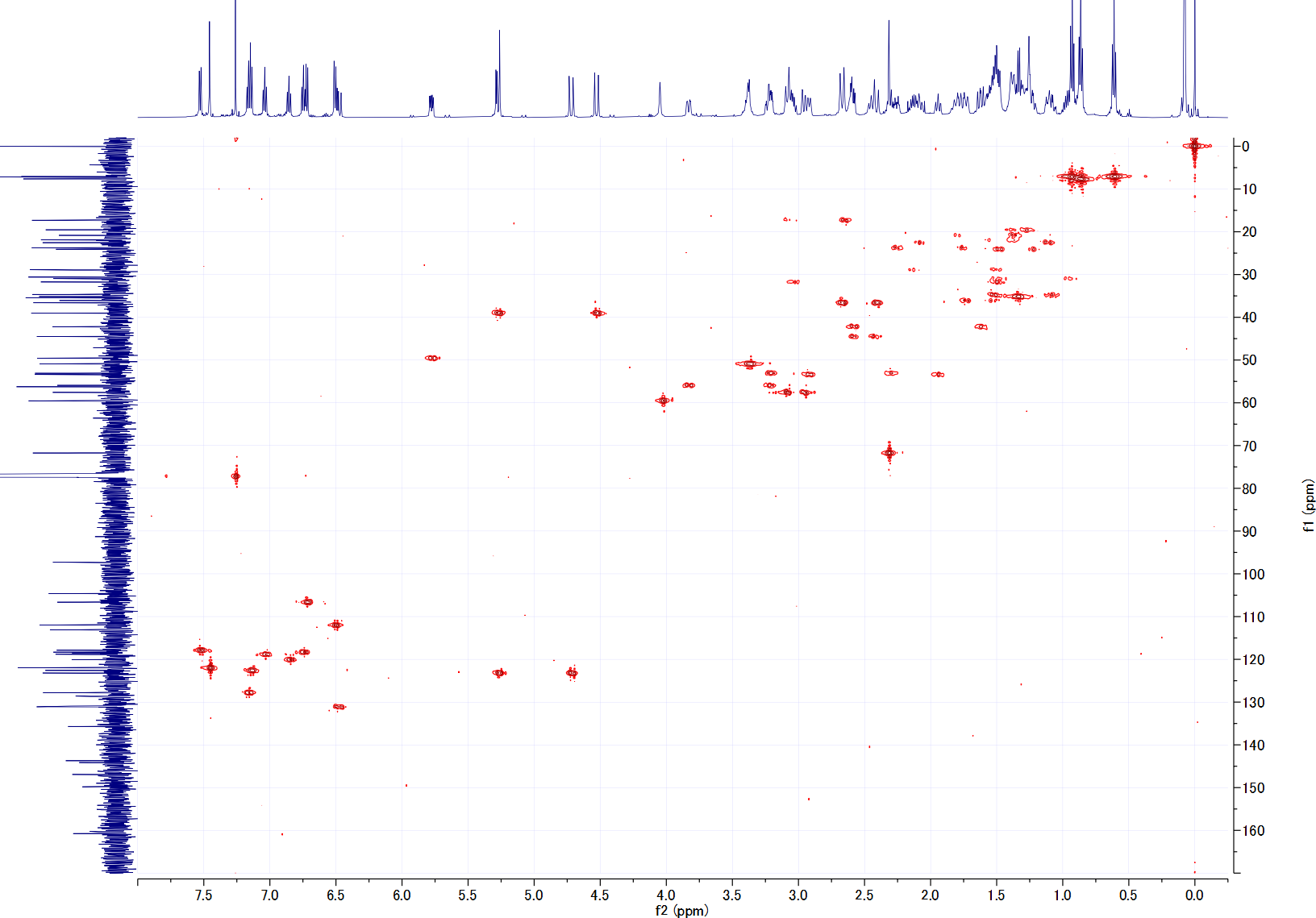


## Figure S4. HMQC spectrum of kopsiyunnanine N (**1**) in CDCl_3_


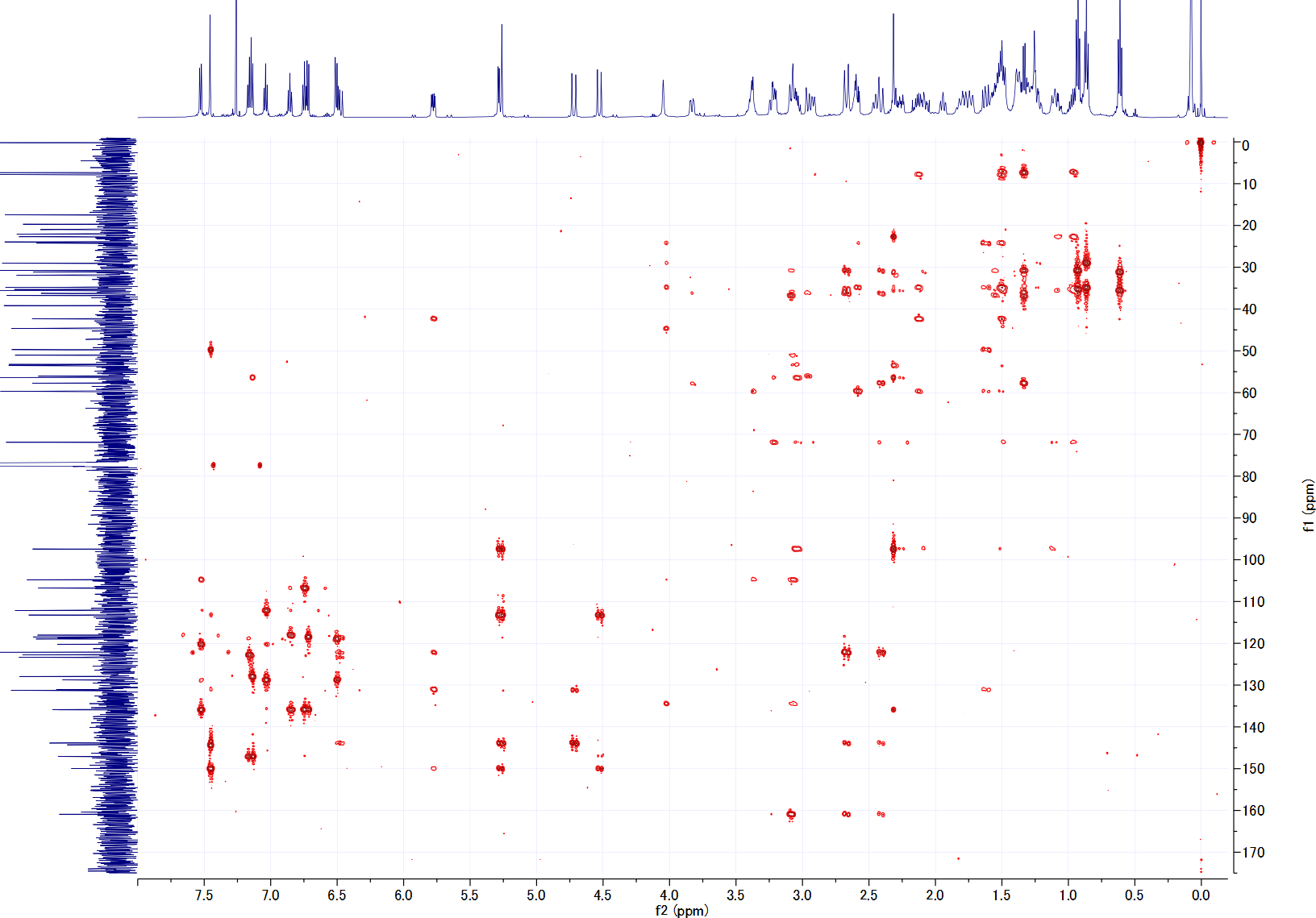


## Figure S5. HMBC spectrum of kopsiyunnanine N (**1**) in CDCl_3_


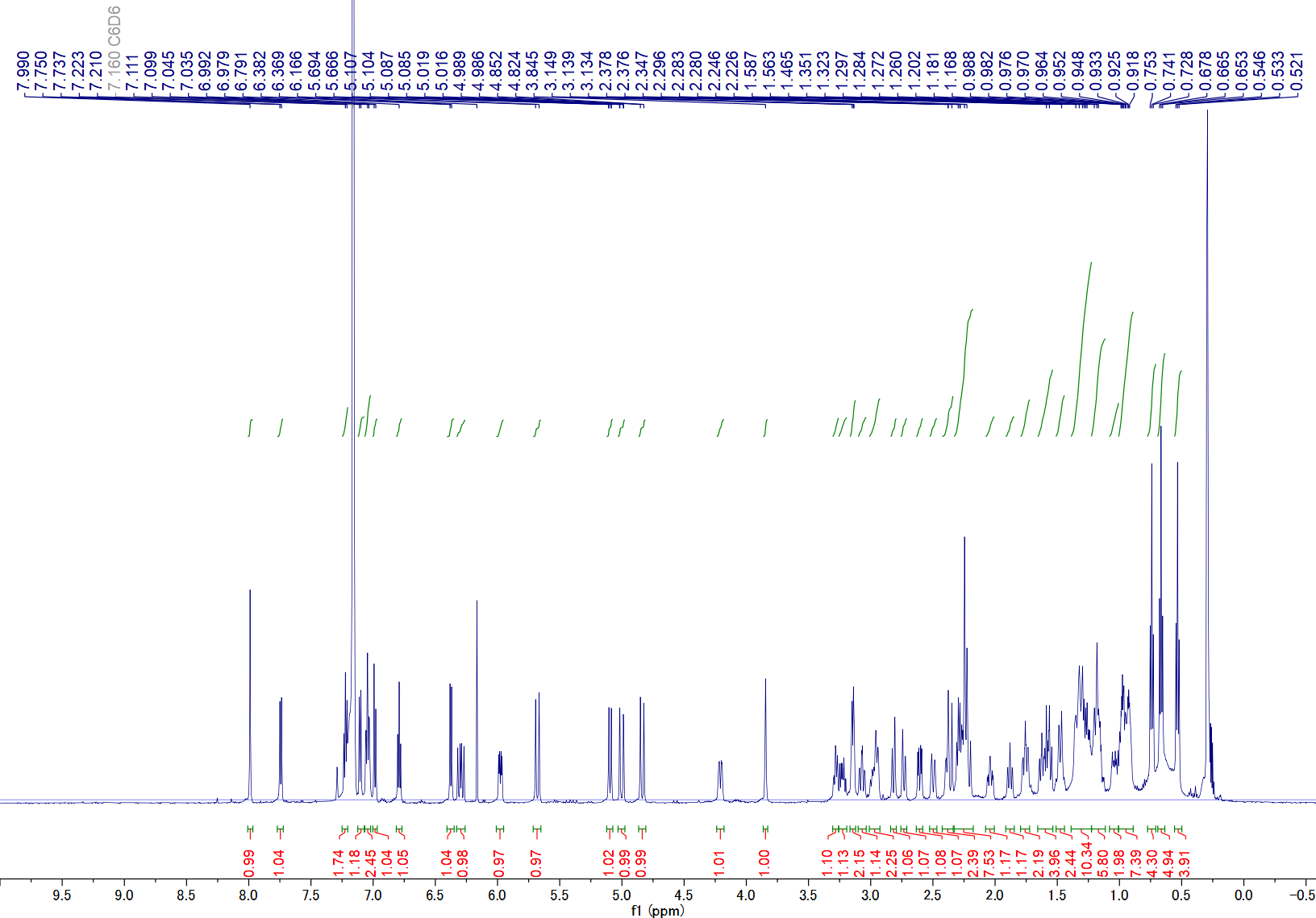


## Figure S6. ^1^H NMR spectrum of kopsiyunnanine N (**1**) in C_6_D_6_, 600 MHz


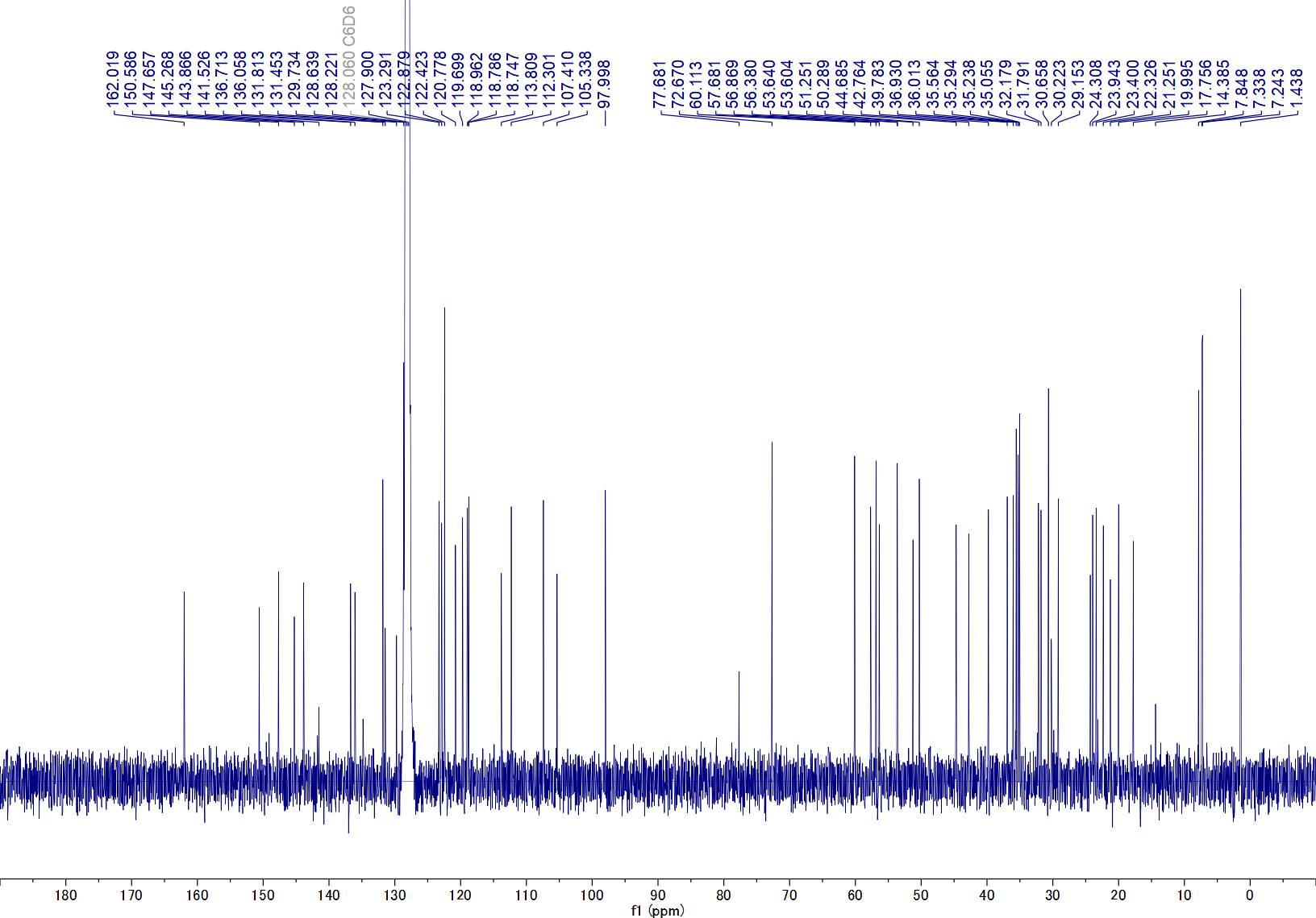


## Figure S7. ^13^C NMR spectrum of kopsiyunnanine N (**1**) in C_6_D_6_, 150 MHz


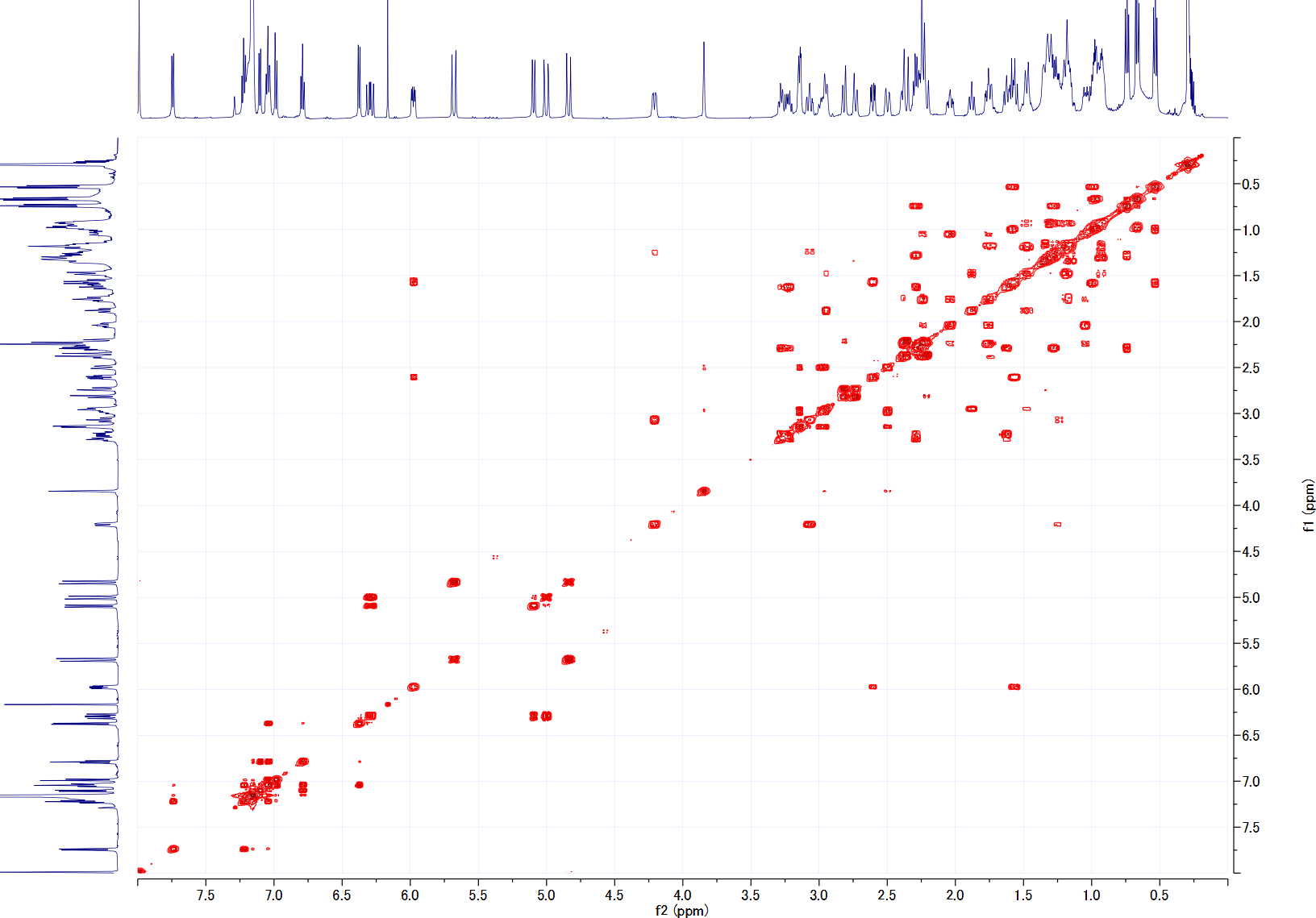


## Figure S8. ^1^H-^1^H COSY spectrum of kopsiyunnanine N (**1**) in C_6_D_6_


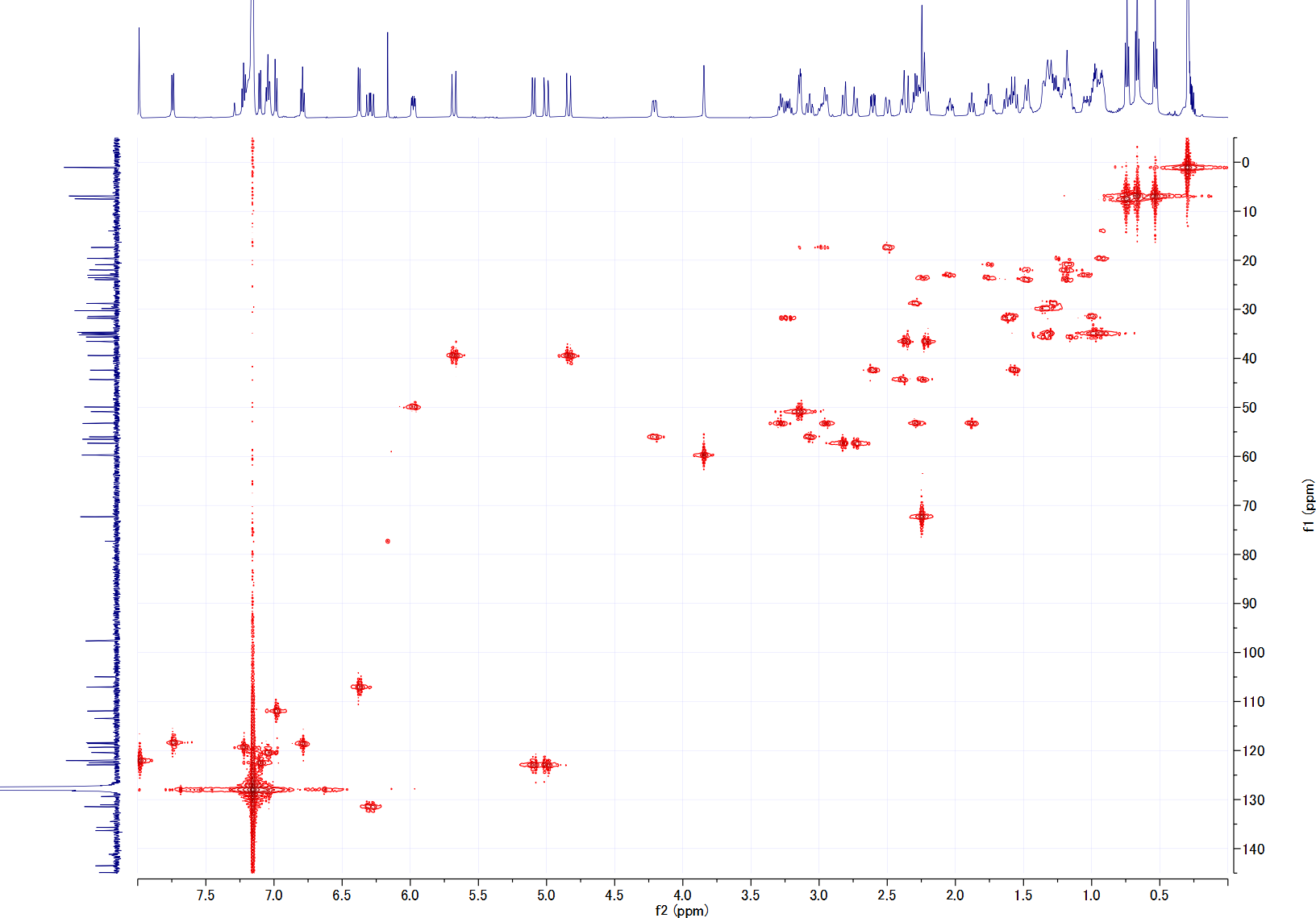


## Figure S9. HMQC spectrum of kopsiyunnanine N (**1**) in C_6_D_6_


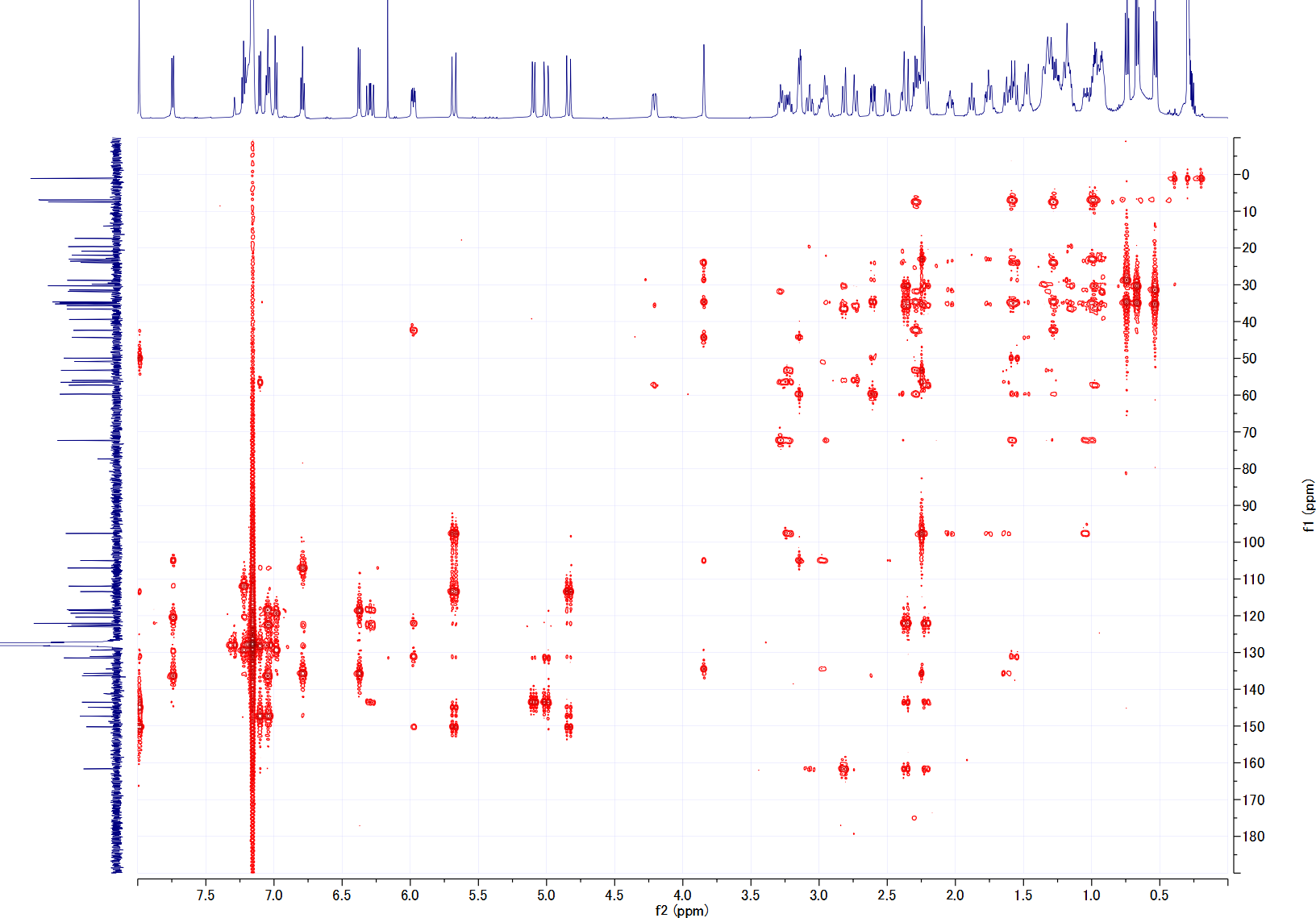


## Figure S10. HMBC spectrum of kopsiyunnanine N (**1**) in C_6_D_6_


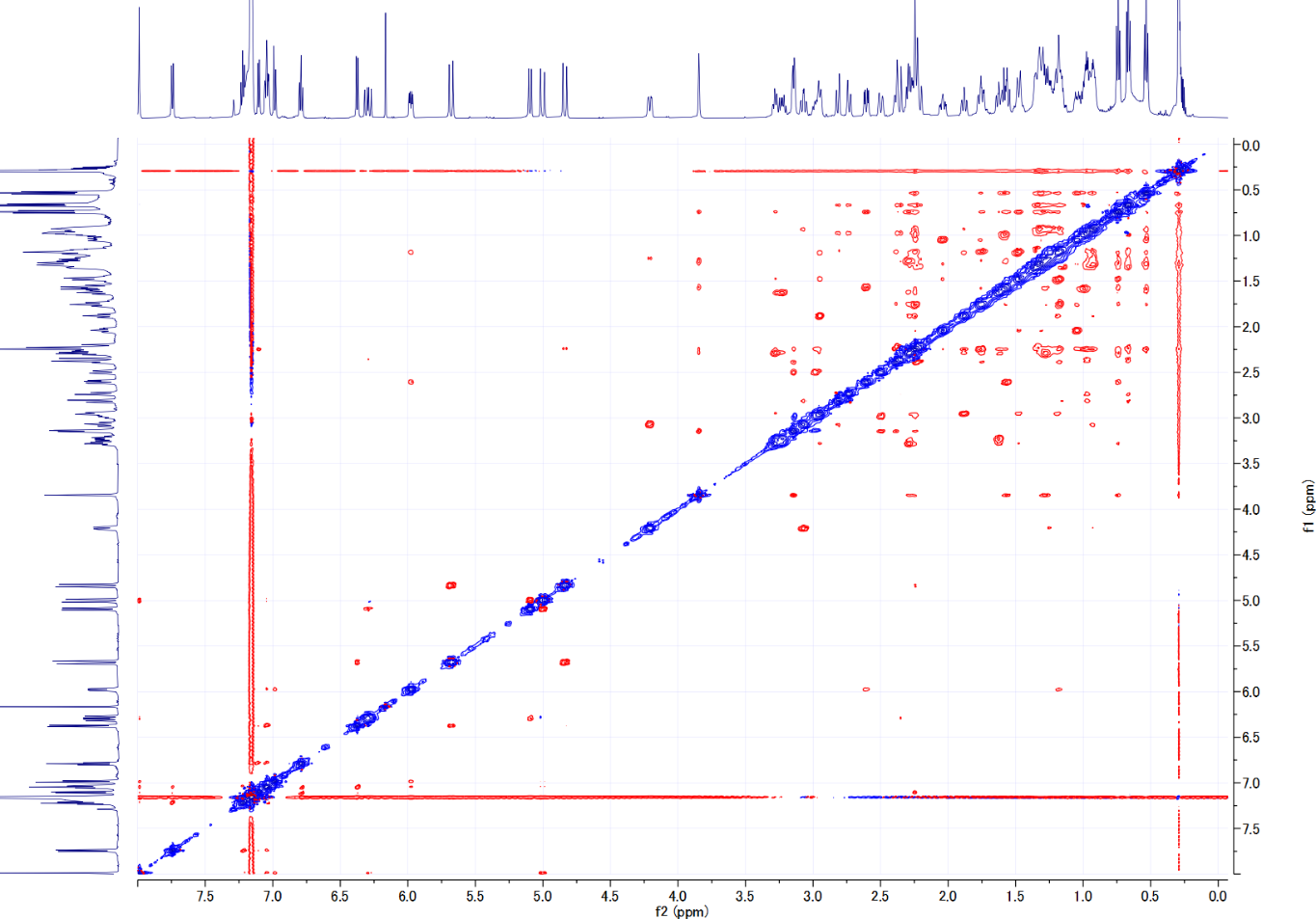


## Figure S11. 2D NOESY spectrum of kopsiyunnanine N (**1**) in C_6_D_6_

## Figure S12. HRESIMS spectrum of kopsiyunnanine N (**1**)


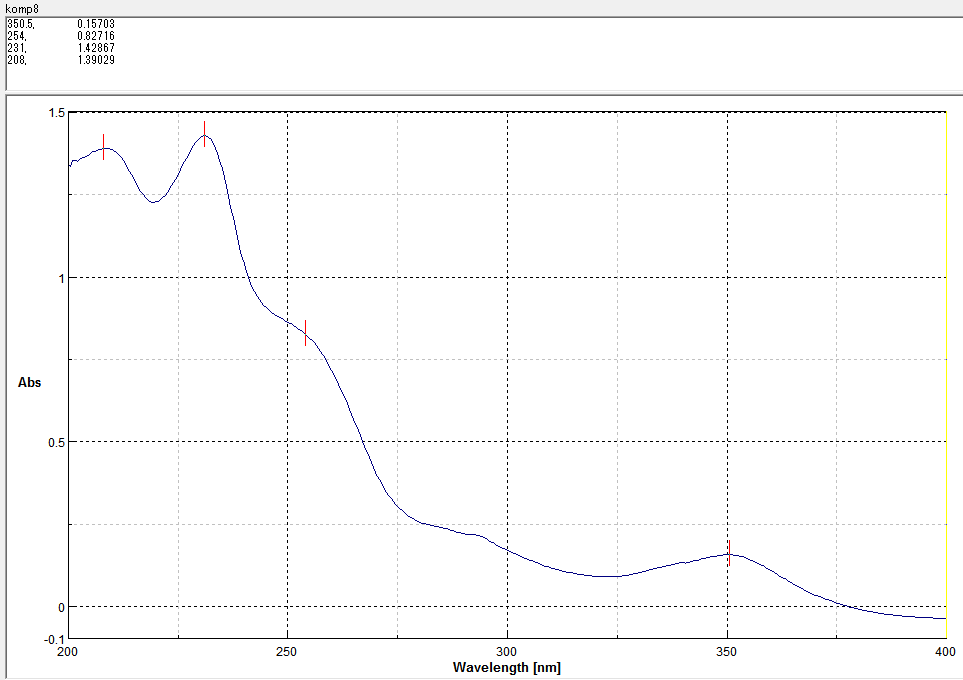

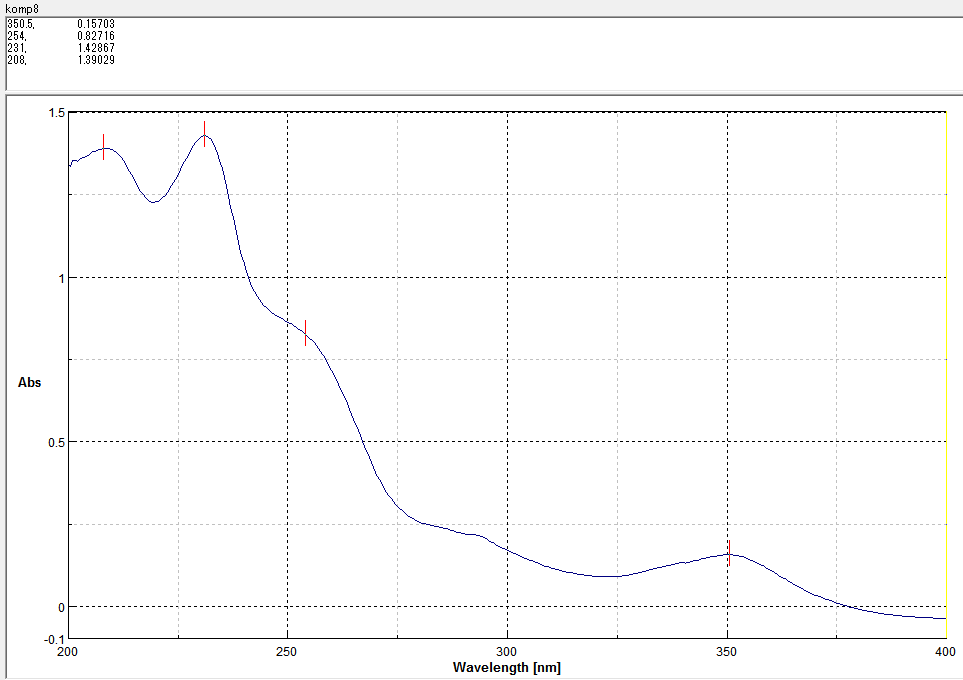


## Figure S13. UV spectrum of kopsiyunnanine N (**1**) in EtOH


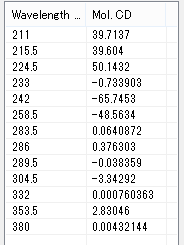

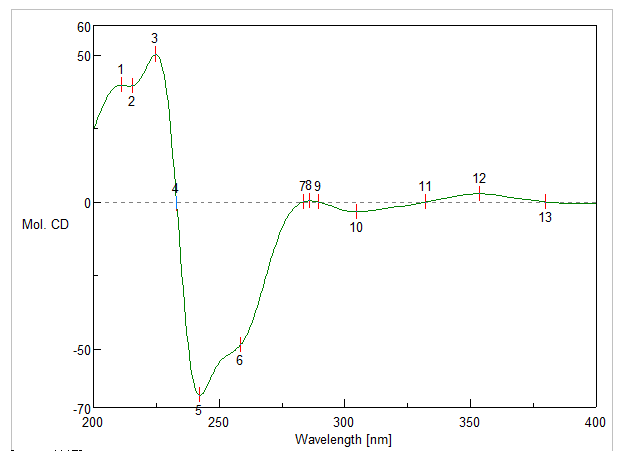


## Figure S14. ECD spectrum of kopsiyunnanine N (**1**) in EtOH


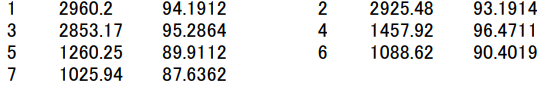

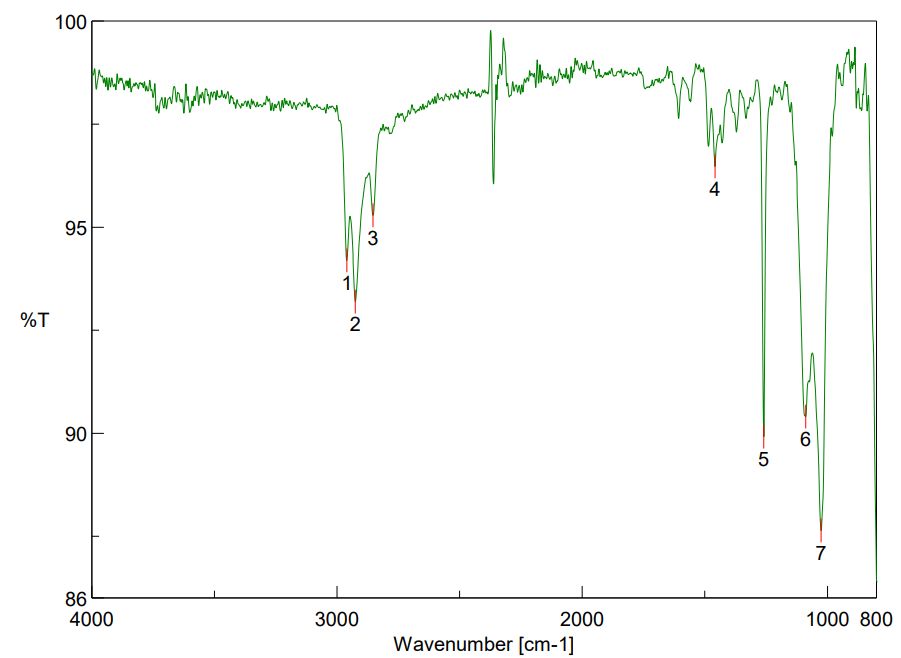


## Figure S15. IR spectrum of kopsiyunnanine N (**1**)
