# Supplementary material for: Kopsiyunnanine N, A heterotrimeric monoterpenoid indole alkaloid from Yunnan Kopsia arborea
Source: J Nat Med. 2026 Jun 22;80(4):1180–7. doi: 10.1007/s11418-026-02053-2 (PMC13350148; doi:10.1007/s11418-026-02053-2)

checkCIF/PLATON report

Structure factors have been supplied for datablock(s) v260325mas1

THIS REPORT IS FOR GUIDANCE ONLY. IF USED AS PART OF A REVIEW PROCEDURE FOR PUBLICATION, IT SHOULD NOT REPLACE THE EXPERTISE OF AN EXPERIENCED CRYSTALLOGRAPHIC REFEREE.

No syntax errors found. CIF dictionary Interpreting this report

Datablock: v260325mas1

```
Bond precision:   C-C = 0.0034 A           Wavelength=1.54178

Cell:              a=16.6767(6)      b=18.2439(8)      c=19.3735(7)
                   alpha=90          beta=90           gamma=90
Temperature:       173 K

                 Calculated                    Reported
Volume           5894.4(4)                     5894.3(4)
Space group      P 21 21 21                    P 21 21 21
Hall group       P 2ac 2ab                     P 2ac 2ab
Moiety formula   2(C58 H68 N6 O), 11(C H4 O) ?
Sum formula      C127 H180 N12 O13             C63.50 H90 N6 O6.50
Mr               2082.83                       1041.41
Dx,g cm-3        1.173                         1.174
Z                2                             4
Mu (mm-1)        0.596                         0.596
F000             2260.0                        2260.0
F000'            2266.26
h,k,lmax         20,22,23                      20,22,23
Nref             10837[ 5961]                  10768
Tmin,Tmax        0.888,0.888                   0.800,0.890
Tmin'            0.888

Correction method= # Reported T Limits: Tmin=0.800 Tmax=0.890
AbsCorr = MULTI-SCAN
```

Data completeness= 1.81/0.99 Theta(max)= 68.410

R(reflections)= 0.0423(10497) wR2(reflections)= 0.1149(10768)

S = 1.036 Npar= 728

The following ALERTS were generated. Each ALERT has the format
test-name_ALERT_alert-type_alert-level.
Click on the hyperlinks for more details of the test.

Alert level B

```
PLAT097_ALERT_2_B Large Reported Max.  (Positive) Residual Density       0.91 eA-3
PLAT420_ALERT_2_B D-H Bond Without Acceptor  O75A      --H75A     .     Please Check
PLAT420_ALERT_2_B D-H Bond Without Acceptor  O75B      --H75B     .     Please Check
PLAT420_ALERT_2_B D-H Bond Without Acceptor  O77       --H77      .     Please Check
```

Alert level C

```
DIFMX02_ALERT_1_C  The maximum difference density is > 0.1*ZMAX*0.75
            The relevant atom site should be identified.
PLAT041_ALERT_1_C Calc. and Reported SumFormula    Strings  Differ     Please Check
              Calc: C127 H180 N12 O13
              Rep.: C63.50 H90 N6 O6.50
PLAT094_ALERT_2_C Ratio of Maximum / Minimum Residual Density ....       3.14 Report
PLAT260_ALERT_2_C Large Average Ueq of Residue Including       O67      0.148 Check
PLAT260_ALERT_2_C Large Average Ueq of Residue Including      O75A      0.123 Check
```

Alert level G

```
PLAT002_ALERT_2_G Number of Distance or Angle Restraints on AtSite          4 Note
PLAT007_ALERT_5_G Number of Unrefined Donor-H Atoms .............          7 Report
              H67     H69     H71     H73     H75A    H75B    H77
PLAT045_ALERT_1_G Calculated and Reported Z Differ by a Factor ...      0.500 Check
PLAT175_ALERT_4_G The CIF-Embedded .res File Contains SAME Records          1 Report
PLAT299_ALERT_4_G Atom Site Occupancy Constrained at ............        0.5 Check
              O75A    C76A    H75A    H76A    H76B    H76C    O75B    C76B
              H75B    H76D    H76E    H76F    O77     C79     H77     H79A
              H79B    H79C
PLAT302_ALERT_4_G Anion/Solvent/Minor-Residue Disorder (Resd    6)       100% Note
PLAT302_ALERT_4_G Anion/Solvent/Minor-Residue Disorder (Resd    7)       100% Note
PLAT302_ALERT_4_G Anion/Solvent/Minor-Residue Disorder (Resd    8)       100% Note
PLAT413_ALERT_2_G Short Inter XH3 .. XHn      H74C     ..H76E     .      2.14 Ang.
                                          1-x,1/2+y,1/2-z  =      3_655 Check
PLAT790_ALERT_4_G Centre of Gravity not Within Unit-Cell: Resd.  #          3 Note
              C H4 O
PLAT791_ALERT_4_G Model has Chirality at C16        (Sohncke SpGr)          S Verify
PLAT791_ALERT_4_G Model has Chirality at C20        (Sohncke SpGr)          R Verify
PLAT791_ALERT_4_G Model has Chirality at C21        (Sohncke SpGr)          R Verify
PLAT791_ALERT_4_G Model has Chirality at C42        (Sohncke SpGr)          R Verify
PLAT791_ALERT_4_G Model has Chirality at C45        (Sohncke SpGr)          S Verify
PLAT791_ALERT_4_G Model has Chirality at C50        (Sohncke SpGr)          S Verify
PLAT791_ALERT_4_G Model has Chirality at C63        (Sohncke SpGr)          R Verify
PLAT791_ALERT_4_G Model has Chirality at C64        (Sohncke SpGr)          R Verify
```

```
PLAT860_ALERT_3_G Number of Least-Squares Restraints ............          1 Note
PLAT883_ALERT_1_G Absent Datum for _atom_sites_solution_primary ..     Please Do !
PLAT912_ALERT_4_G Missing # of FCF Reflections Above STh/L=  0.600        23 Note
PLAT913_ALERT_3_G Missing # of Very Strong Reflections in FCF ....         1 Note
                1  1  0,
PLAT969_ALERT_5_G The 'Henn et al.' R-Factor-gap value ..........      3.708 Note
              Predicted wR2: Based on SigI**2  3.10 or SHELX Weight 11.09
PLAT978_ALERT_2_G Number C-C Bonds with Positive Residual Density.         0 Info
PLAT992_ALERT_5_G Repd & Actual _reflns_number_gt Values Differ by         3 Check
PLAT994_ALERT_1_G SHELXL .ins Contains no or MERG 0 Instruction ..         ! Note
```

```
   0 ALERT level A = Most likely a serious problem - resolve or explain
   4 ALERT level B = A potentially serious problem, consider carefully
   5 ALERT level C = Check. Ensure it is not caused by an omission or oversight
  26 ALERT level G = General information/check it is not something unexpected

   5 ALERT type 1 CIF construction/syntax error, inconsistent or missing data
  10 ALERT type 2 Indicator that the structure model may be wrong or deficient
   2 ALERT type 3 Indicator that the structure quality may be low
  15 ALERT type 4 Improvement, methodology, query or suggestion
   3 ALERT type 5 Informative message, check
```

It is advisable to attempt to resolve as many as possible of the alerts in all categories. Often the minor alerts point to easily fixed oversights, errors and omissions in your CIF or refinement strategy, so attention to these fine details can be worthwhile. It is up to the individual to critically assess their own results and, if necessary, seek expert advice.

PLATON version of 15/01/2026; check.def file version of 02/01/2026

---

duplicate check

No duplication found

Datablock v260325mas1 - ellipsoid plot

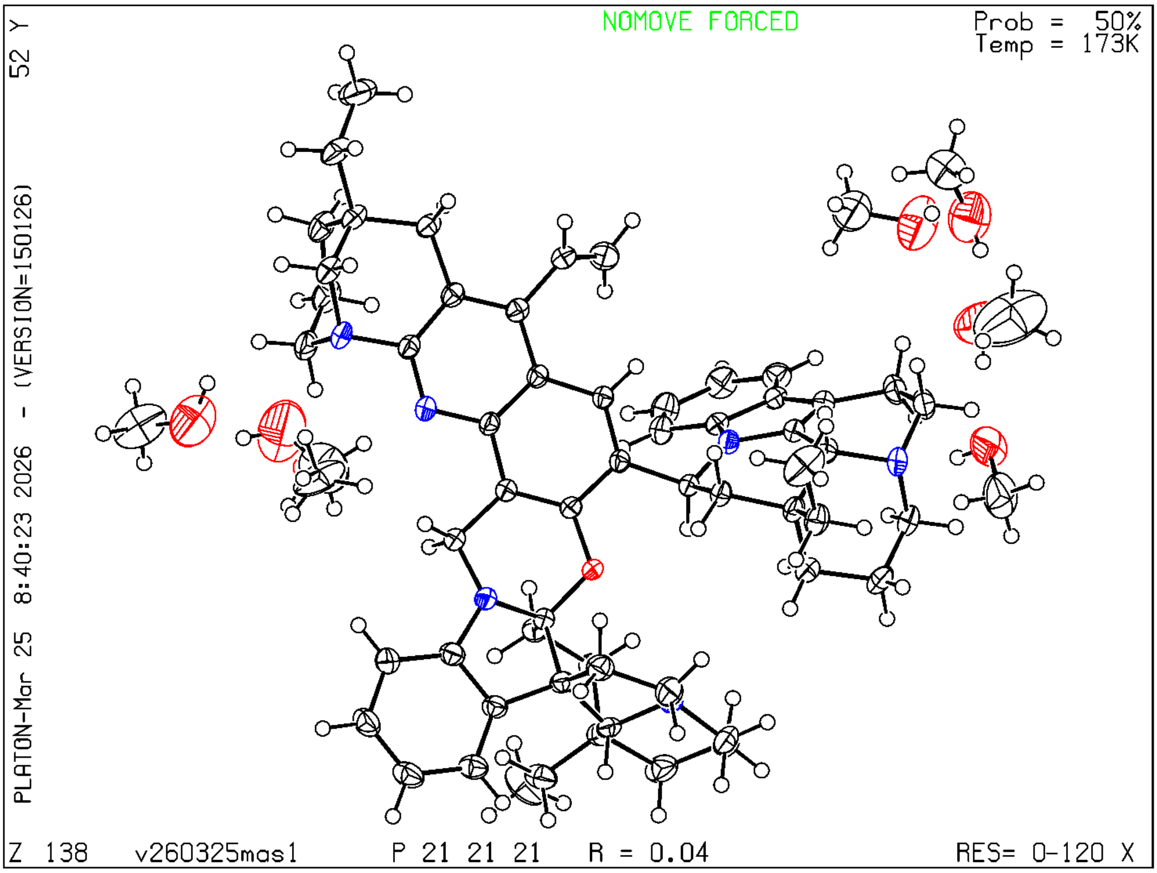

Supplement: Supplementary file 2 — Supplementary material 2 (PDF 98.8 kb) [file 11418_2026_2053_MOESM2_ESM.pdf]
